# Estimating the broader fiscal consequences of acute hepatic porphyria (AHP) with recurrent attacks in Belgium using a public economic analytic framework

**DOI:** 10.1186/s13023-021-01966-3

**Published:** 2021-08-04

**Authors:** Mark P. Connolly, Nikos Kotsopoulos, Sebastian Vermeersch, Julien Patris, David Cassiman

**Affiliations:** 1 Global Market Access Solutions Sarl, St-Prex, Switzerland; 2 Unit of Pharmacoepidemiology and Pharmacoeconomics, Department of Pharmacy, University of Groningen, 9713 AV Groningen, The Netherlands; 3 HICT, Ottergemsesteenweg-Zuid 808 B/354, 9000 Ghent, Belgium; 4 Alnylam Pharmaceuticals, Antonio Vivaldistraat 150, 1083 HP Amsterdam, Netherlands; 5 Metabolic Center, Department of Gastroenterology-Hepatology, University of Leuven, Leuven, Belgium

**Keywords:** Acute hepatic porphyria (AHP), Disability, Public economics, Cost-analysis, Taxation, Public benefits, Employment, Fiscal analysis

## Abstract

**Background:**

Acute hepatic porphyria (AHP) is a rare, debilitating disease characterized by potentially life-threatening attacks often resulting in chronic symptoms that negatively impact daily functioning and quality of life. Symptoms of AHP prevent many individuals from working and achieving lifetime work averages. The aim of this study was to apply a public economic framework to evaluate AHP in Belgium, taking into consideration a broad range of costs that are relevant to government in relation to social benefit payments and lifetime taxes paid.

**Methodology:**

A public economic framework was developed exploring lifetime costs for government attributed to an individual with AHP and recurrent attacks in Belgium. Work-activity and lifetime direct taxes paid, indirect consumption taxes and requirements for public benefits were estimated based on established clinical pathways for AHP and compared to the general population (GP). The model includes AHP-related healthcare costs and non-AHP healthcare costs for the GP.

**Results:**

Lifetime earnings are reduced in an individual with AHP by €347,802 per person (p.p.), translating to reduced lifetime taxes paid of €183,187 for an AHP individual compared to the GP. We estimate increased lifetime disability benefit support of €247,242 for an AHP individual compared to GP. Lifetime healthcare costs for a person with AHP were estimated to be €3,030,316 due to frequent hospitalisations associated with porphyria attacks compared to the GP. The lifetime costs for a person with 12 attacks per annum factoring in transfers, taxes and healthcare costs are estimated to be €3,460,745 p.p. Eliminating AHP attacks after 10 years of active disease, thus, enabling a person to return to work increases lifetime earnings by €224,575 p.p. Increased work activity in such individuals would generate an estimated €118,284 p.p. over their lifetime. The elimination of AHP attacks could also lead to reductions in disability payments of €179,184 p.p. and healthcare cost savings of €1,511,027 p.p.

**Conclusions:**

Due to severe disability resulting from constant attacks, AHP patients with recurrent attacks incur significant public costs. Lifetime taxes paid are reduced as these attacks occur during peak earning and working years. In those patients, reducing AHP attacks can confer significant fiscal benefits for government, including reduced healthcare costs, reduced disability payments and improved tax revenue.

**Supplementary Information:**

The online version contains supplementary material available at 10.1186/s13023-021-01966-3.

## Background

Acute hepatic porphyria (AHP) is a group of 4 ultra-rare, genetically distinct diseases that are characterised by episodes of neuro-visceral attacks, with occasional cutaneous manifestations [1]. The 4 diseases of AHP are acute intermittent porphyria (AIP), variagate porphyria (VP), hereditary coproporphyria (HCP), and hereditary deficit of delta-aminolevulinic acid dehydratase (ALA dehydratase-deficiency porphyria (ADP). AHP are disorders that result from a genetic defect leading to deficiency in one of the enzymes of the haeme precursors biosynthesis pathway in the liver [2, 3]. All AHP subtypes have a common pathophysiology caused by a genetic mutation leading to a partial deficiency in the activity of one of the enzymes responsible for heme synthesis [4]. In acute porphyrias, the respective enzyme deficiencies predispose patients to a variety of triggering factors, which provoke the accumulation of the neurotoxin (aminolevulinic acid synthase (ALA) and porphobilinogen PBG) [3]. Being neurotoxins, both ALA and PBG are harmful to nerve cells and are associated with the episodes of neuro-visceral attacks.

Various agents and physiological factors can trigger an episode including excess alcohol, certain drugs, such as anticonvulsants, oral contraceptives, sulfonamides, cytochrome P450 inducers, rapid weight loss, acute illness and infection, stress, hormonal factors related to the luteal phase of the menstrual cycle as well as pregnancy and the postpartum period [3]. Symptoms vary from one form of AHP to another, and can include symptoms influencing QoL including severe pain, respiratory failure, seizures, hallucinations, anxiety, weakness, fatigue, numbness, nausea and vomiting, lesions or blistering on sun-exposed skin [5]. Patients presenting at hospital may have many symptoms related to dysfunction in the autonomic, central and peripheral nervous systems [6].

People with AHP experience a significant impairment of their health-related quality of life as they can suffer frequent and/or severe attacks [7] as well as experience chronic symptoms between attacks [8]. Not all AHP patients will experience recurrent acute attacks and/or chronic debilitating symptoms. Some studies estimate the percentage of symptomatic patients developing recurrent acute attacks between 3–5% and 10% [9–11].

There is limited data characterizing the disease in severely affected patients but the burden of disease of AHP with recurrent attacks has been documented by a natural history study conducted in 112 AHP patients who experience recurrent attacks, defined at 3 or more porphyria attacks within the 12 months prior to the start of the study. Sixty-five percent of the patients reported experiencing chronic symptoms, 20% experienced 6 to 10 attacks in the preceding 12 months, while as many as 32% reported having suffered more than 10 attacks in the preceding 12 months [6]. Furthermore, AHP is associated with serious long-term, health-limiting complications, including liver disease such as cirrhosis and cancer, chronic kidney disease and systemic arterial hypertension [6]. A patient-level data analysis of 88 patients observed that patients with recurrent porphyria attacks (n = 11) and those who were symptomatic (n = 24) experienced multiple chronic comorbidities. Of those rated as “recurrent cases”, 72.7%, 63.6% and 9.1% also had hypertension, chronic kidney disease and hepatocellular carcinoma, respectively [12].

The attacks and complications related to AHP can lead to absenteeism from work, or even an inability to work. In the natural history study by Gouya et al.(2020), of those who were employed (part-time or full-time), 52% missed work due to their porphyria, with a mean of 40.2 day’s work missed, and at least 21% of the patients had received disability allowances because of their porphyria in the past 12 months [6]. In the cohort data analysis by Neeleman et al. (2018), 63.6% and 33.3% were unemployed in the recurrent cases and symptomatic group, respectively [12].

Current evidence suggests that there is significant unmet need in the treatment of AHP with recurrent attacks which results in substantial burden to patients, absenteeism and loss of productivity, and the associated financial burden for the healthcare system. This study sought to apply a public economic framework taking into consideration a broad range of costs that are relevant to government in relation to transfer payments and taxes paid by people with AHP. Specifically, the evaluation considers both direct and indirect taxes applied to lifetime earnings. For those unable to work due to AHP clinical burden, we apply the standard disability pension for individuals in Belgium. As most healthcare costs in Belgium are tax financed, we also include direct health costs as these are paid by government. The model then uses clinical scenarios to compare with the general population to illustrate how people with AHP will vary compared to the general population based on lifetime taxes paid and transfers received. The analysis described here is not intended to serve as a burden analysis for all AHP patients. Rather, the analysis aims to highlight the broader costs of the disease when considering its multisystemic and chronic nature, as well as the broader impact on the individual and society. By doing so, the authors wish to contribute to the discussion on the broader economic and social impact of rare and ultra-rare disease, and the societal value of addressing those needs despite the rarity of the conditions.

## Methods

### Analytic framework

A public economic model was developed to assess lifetime tax contributions and government payments received for disability, pensions and healthcare for a person with AHP compared to the GP in Belgium. In this regard the main perspective explored is the “government perspective” and how changes in health influence tax revenue for government and payments. We also report societal costs, but this is not the main focus of our work. The analytic framework is similar to methodologies used by governments to assess the impact of policy changes on public accounts; and, in the present context, how different health conditions influence government accounts [13, 14]. The model was used to project the life course of people with AHP compared to the GP taking into consideration workforce participation, age-specific earnings and retirement at age 65. The model annually adjusts for age-specific disability in the GP and mortality in AHP and GP populations using published life tables for Belgium [15]. The model was developed to capture lifetime direct and indirect taxes and transfer payments from the government in each year of the model while adjusting for mortality.

### Wages and taxes

Lifetime direct taxes were derived from age-specific annual earnings obtained from Statbel [16], and inflated based on the percentage increase in reported taxable income over the period of 2011–2017 [17]. Taxes were estimated by applying the age-specific gross earnings adjusted for the age-specific economic activity rate and multiplied by the tax wedge (the difference between before-tax and after-tax wages) to estimate annualised direct tax contributions and adjusting for mortality [16, 18, 19]. Indirect taxes were estimated by applying the 21% VAT rate to the proportion of age-specific disposable income to gross earnings [20]. The direct and indirect taxes combined represent the gross tax contributions (Table 1).

**Table 1:** Fiscal parameters applied in public economic assessment of AHP

Parameter	Input value	Source
Average annual retirement pension	€14,400	[31]
Average annual disability payment	€13,613	[20]
Average annual wage growth	1.3%	[17]^a^
Average annual cost inflation	0.62%	[22]
Average annual healthcare inflation	2%	[32]
Tax wedge	52.7%	[18]
Value added tax	21%	[33]

^a^Derived from net taxable income reported over period 2011–2017

### Disability status

Disability costs for being unable to work due to AHP were compared to background disability in the GP. The impairment imposed by AHP on work activity was adjusted according to severity of AHP, where the unemployment rate has been reported as 63.6% and 33% for recurrent and symptomatic AHP, respectively [12]. In our analysis, we considered that 100% of those with recurrent AHP would be classified as disabled, and 50% of those with symptomatic AHP are unable to work. The age-specific disability rate in the GP was obtained from the European Union Statistics on Income and Living Conditions (EU-SILC) provided by Statistics Belgium and applied to work activity in the GP [20]. The average annual disability transfer payment obtained from household survey data was applied for every year of disability and adjusted for mortality using Belgium life tables [20, 21] (Table 1). All transfer payments were increased annually according to the rate of inflation in Belgium [22].

### AHP clinical scenarios

Disease manifestation and progression are heterogenous in people with AHP, therefore limited data sets are available for defining a cohort on which to model. Therefore, to reflect the broader impact of recurrent attacks, clinical practice and validation of the associated costs was obtained from expert opinion and was validated with finding in the literature as part of establishing the healthcare resource use [23]. To reflect the heterogeneity of the disease, we developed three scenarios to reflect fiscal costs to government based on likely clinical scenarios. Each scenario corresponds to a plausible scenario for an AHP patient experiencing recurrent attacks comprised of an episode of attacks that either continues from onset or ends after a period of time and varying impact on ability to work. On a population level, the recurrent attack population likely comprises a mix of these and other types of patients, and we do not consider any of these scenarios to be the dominant case. For each scenario we estimated the likely fiscal consequences associated with the condition for a single individual, i.e., cohort n = 1. In all cases, we considered that AHP symptom onset was aged 30 with an average of 12 attacks per year and an analytic time horizon set to age 100 and compared with a non-AHP individual in the general population. In all three scenarios the costs were counted from age 30 onwards.

- **Scenario 1**: AHP attacks throughout life and unable to work (AL-W)
- **Scenario 2:** AHP attacks for 10 years, stopping at age 40, unable to work due to chronic comorbidities attributed to AHP (A10-W) (used in sensitivity analysis)
- **Scenario 3**: AHP attacks for 10 years, stopping at age 40, able to return to work (A10 + W).

A diagram depicting the three scenarios has been included in the Additional file 1.

### Health costs

Health costs for people with AHP included three components: 1) AHP treatment management costs; 2) costs of treating AHP-attributable complications; 3) cost per hospitalized AHP attack. Annual age-specific healthcare expenditure for non-AHP individuals in the GP (GP) adjusted for inflation have been included for comparison [24]. Costs were estimated on a micro-costing approach of multiplying resource use by the Belgian unit cost, and supported with costs obtained from the literature, and adjusted to 2020 costs. Hospitalization costs were calculated according to Belgian guidelines [25], taking into account an average daily cost per hospitalization, a daily lump sum for drugs, a per stay lump sum for clinical biology and a per stay lump sum for medical imaging. The cost per attack that was treated in hospital was calculated by feedback on resource use from the expert consultation process [23], with unit cost data extracted from the national web-based pricelists [26, 27] (Table 2).

**Table 2:** Average cost per AHP attack treated in hospital

Resource	% receiving	Units used	Unit cost	Total cost	Source
Opioids (morphine)	100	18^a^	€0.68	€12.29	[23]
Hemin	100	4^b^	€592.10	€2,368.39	[23]
Hemin side effects	100			€102.45	Additional file 1: Table S1
Albumin	100	4	€29.63	€118.52	[23]
Hospital admission, ER	100	1	€43.69	€43.69	[23]
Hospital admission, ICU	20	2	€179.69	€69.08	[23]
Hospitalization days	100	7	€492.27	€3,440.42	[23, 25]
Blood test	100	1	€26.00	€26.00	[23]
Total				€6,180.83	

^a^Example, morphine 5 mg hourly as required for 36 h

^b^ Normosang 3 mg/kg once daily for 3 days

Hemin treatment is associated with several serious (grade 3 or 4) adverse events. The cost for treating these adverse events is calculated as an additional cost during the hospitalization for an acute attack and considers only an extension of the hospitalization period by 3–5 days [23]. The details of costs per event are provided in the Additional file 1: Table S1.

The average annual AHP-related cost per co-morbidity or chronic disorder and AHP-related acute attacks were derived from the literature and adjusted to 2020 values where necessary (Additional file 1: Table S2). Disease management and supporting costs per year were calculated by multiplying the annual cost by the proportion of patients with comorbidities per annualised attack rate (AAR) state, taking into account the distribution of patients across the different AAR categories [12] as shown in Additional file 1: Table S3. The resulting annual costs excluding social costs per category are: € 6,192 (asymptomatic), € 20,930 (symptomatic), € 34,252 (recurrent) and € 34,252 (severe).

### Analytic output

For each scenario, the model estimates the amount of transfers received every year and over the lifetime, which include disability payments, pension costs and public health costs, in order to derive total transfers. The output estimates the societal costs from lost productivity applying the human capital approach. The model also estimates the amount of lifetime taxes paid in each scenario based on age-specific average earnings adjusted for mortality and applied to the Belgian tax wedge [18]. All results presented are based on a single individual and represent the per person (p.p.) costs. Costs have been discounted annually at 3% as per guidelines on cost-effectiveness analysis in Belgium [28].

### Sensitivity analysis

A one-way sensitivity analysis was performed on several critical parameters that influence fiscal consequences for government. It is not possible to claim one of the scenarios in our analysis as the dominant base case, however we have selected Scenario 2 as the basis for conducting the sensitivity analysis to reflect variability. In the sensitivity analysis we explore variation in the annual number of attacks, duration of attacks in years, the influence of chronic health costs, the cost per AHP attack, and annual disability payment for those out of work. We present variability in the sum of government costs in a tornado diagram applying ± 25% variation to all parameters.

## Results

**Fig. 1:**
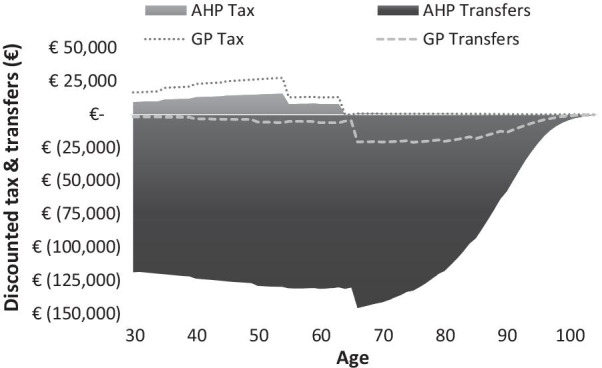
Impact of AHP on government transfers and taxes attributed to a person diagnosed at age 30 and experiencing 12 attacks per year over lifetime (discounted 3%)

The model estimates the impact of AHP on government cash flow from taxes collected and costs associated with healthcare and disability support pensions. These are reflected graphically demonstrating how the per capita transactions change every year. The model starts at age 30 at the point of diagnosis and counts differences in taxes (positive) between AHP and average GP person. Additionally, the model calculates government costs (negative) over time for different public programs (e.g., disability, healthcare, pensions) while adjusting for mortality using Belgian life tables. We present the figure for scenario 1 which demonstrates the impact of AHP attacks throughout life and unable to work (AL-W) in Fig. 1, and the figures for scenarios 2 and 3 are provided in the Additional file 1.

The dominant costs for government associated with AHP are due to healthcare spending on attacks and associated comorbidities. Those experiencing lifetime AHP attacks of 12 per year incur €3,100,534 in discounted healthcare expenses compared to €70,218 in the GP resulting in an additional €3,030,316 costs over the lifetime of the individual (Table 3). When attacks are stopped after 10 years, the lifetime healthcare discounted costs are estimated to be €1,519,284 p.p. more compared to the GP (€70,218 p.p.). The lifetime taxes paid was lower in those unable to work due to AHP (€251,535 p.p.) resulting in lost tax revenue of €183,187 for AL-W and A10-W compared to GP (€434,722 p.p.). For those individuals able to return to work without permanent disability in Scenario 3 (€369,819 p.p.), reductions in tax of €64,903 p.p. would be anticipated compared to the GP (€434,722 p.p.).

**Table 3:** Government costs and lost tax revenue for three different AHP clinical scenarios (discounted at 3%)

	GP	Scenario 1 AL-W	Change 1 (AL-W – GP)	Scenario 2 A10-W	Change 2 (A10-W – GP)	Scenario 3 A10 + W	Change 3 (A10 + W – GP)
Disability transfers	€40,277	€287,519	€247,242	€287,519	€247,242	€108,335	€68,058
Pension costs	€83,110	€83,110	€0	€83,110	€0	€83,110	€0
Health costs	€70,218	€3,100,534	€3,030,316	€1,589,502	€1,519,284	€1,589,502	€1,519,284
Sum of government costs	€193,605	€3,471,163	€3,277,558	€1,960,131	€1,766,526	€1,780,947	€1,587,342
Lifetime earnings	€632,367	€284,565	− €347,802	€284,565	− €347,802	€509,141	− €123,226
Gross tax	€434,722	€251,535	− €183,187	€251,535	− €183,187	€369,819	− €64,903
Work years	25.24	11.36	− 13.88	11.36	− 13.88	20.88	− 4.36

AL-W, AHP attacks throughout life and unable to work

A10-W, AHP attacks for 10 years, stopping at age 40, unable to work due to chronic comorbidities attributed to AHP

A10 + W, AHP attacks for 10 years, stopping at age 40, able to return to work

Improved working years were assessed in AHP individuals (A10 + W) that reduced AHP attacks. The improved work activity in this group generated lifetime earnings of €509,141 compared to those unable to work of €284,565 p.p. The improved work activity after reducing attacks can generate €369,819 p.p.in lifetime tax contributions compared to €251,535 p.p. in those people with AHP not able to return to work.

### Sensitivity analysis

**Fig. 2:**
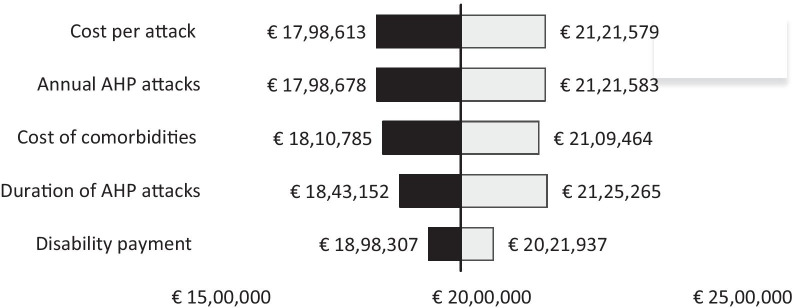
One-way sensitivity analysis (OWSA) based on ± 25% variation in input parameters applied to scenario 2

Healthcare costs are the dominant costs in all scenarios explored. As noted in our sensitivity variation in cost of treatment per attack and annual AHP attacks had similar impact on the sum of all government costs. Varying disability payments for those unable to work had the least impact on fiscal variability (Fig. 2).

## Discussion

Many chronic health conditions and acute medical events can lead to economic consequences that extend beyond health service costs. People with AHP suffer from impaired physical functioning and ongoing symptoms between attacks, of which chronic pain is the most notable and frequently observed, in addition to mood disorders and gastrointestinal symptoms [3]. Many people manage chronic pain with regular opioid use, thereby putting themselves at risk of opioid dependency [9]. Due to the symptoms associated with acute attacks and underlying comorbidities associated with AHP can lead to employment disruptions and in many cases cause people to withdraw from work entirely and influence quality of life. This not only influences the earning capacity of individuals which can influence households but can also influence the amount of lifetime taxes paid and the need for social benefit support provided by governments. In our analysis we capture the fiscal consequences on government associated with AHP.

Treatment practices have traditionally focused on treating acute AHP attacks. Acute AHP attacks can be treated with prompt intravenous hemin (an iron-containing porphyrin) therapy to avoid the development of potentially irreversible neurologic effects. The goal of treatment in acute attacks is to reduce the activity of hepatic 5-aminolevulinic acid synthase 1. Other treatment approaches for AHP include carbohydrate loading and intravenous glucose which can be administered during an attack or sometimes between attacks (prophylactically). However, hemin is not currently approved for the prophylactic treatment of AHP [1, 3]. Additionally, patients may require symptomatic treatment of autonomic dysfunctions, sensorimotor neuropathy and encephalopathy, as well as exclusion of the causative factors and adequate nutrition and fluid therapy [29].

Recently new therapies have been launched that have been shown to influence the number of attacks [30]. The analysis described here illustrates how preventing AHP attacks and likely changing the life course of people with AHP can offer public economic benefits for government. As described here, preventing disability in one person with AHP can save government approximately €247,242 per person in projected disability payments. The broader fiscal analysis described here can help health technology assessment (HTA) agencies understand the broader value of treatment, and provide inputs for cost-effectiveness analysis.

There are several weaknesses of the modeling approach described here. Firstly, to reflect the heterogenous nature of AHP, a scenario-based modeling approach was applied compared to a cohort model that would rely on statistical norms for disease outcomes and disease progression. Furthermore, due to limited information on AHP associated mortality, we applied the average life-expectancy to the AHP individuals. This may be an over-estimate of current life-expectancy. Early mortality in people with AHP would have an impact on government public accounts by reduced spending on healthcare and disability benefits. Additionally, the fiscal modeling framework does not include the option for liver transplantation which is common in people with AHP that can improve outcomes, however it would also increase medical costs with only limited influence on the likelihood of returning to work. Finally, one of the major cost drivers in the analysis relates to how AHP influences work force participation and whether people discontinue work or not. In two of our scenarios, we assumed that AHP would cause people to withdraw permanently from the work force and in the third scenario we assumed 10 years of employment inactivity. This may not reflect the actual employment trajectory of people with AHP, but, in this regard, our work reflects the likely benefits for government from preventing disease progression and keeping people in the work force.

The fiscal analysis described here relies heavily on the impact that AHP can have on labour market activity and reliance on public benefits to maintain living standards i.e. disability. As AHP can manifest in many ways and influence an individuals’ employment trajectory, there are some simplifications applied that may overestimate fiscal gains and losses. For example, in the scenario with people returning to work, we have estimated average earnings based on age-specific wages for Belgium for person returning to work. However, this may be an overestimate as the person with AHP may return to part-time work. Additionally, as they have been out of the work force, they may not achieve normal age-specific wages as they will likely have less experience compared with similar aged workers resulting in lower wages. Furthermore, we were unable to obtain age-specific disposable income in Belgium that deducts the anticipated savings rate. As such, our estimates of indirect taxes assume full consumption of disposable income which may not be the case. Additionally, in some individuals, symptom onset can occur in puberty which can influence learning and educational attainment. This would negatively influence future earnings due to reduced educational attainment which has not been considered as a scenario in our analysis. The diversity of potential life-trajectories described here illustrates the complexity of trying to reflect a homogenous cohort of individuals with AHP and why we opted to reflect clinical scenarios.

## Conclusion

The constant attacks associated with AHP designate this condition as a severe disability and cause significant public costs. As these attacks occur during peak earning and working years, work activity and lifetime taxes paid are reduced. Decreasing AHP attacks can present significant fiscal benefits for government, including reduced health costs, reduced disability payments and improved tax revenue.

## Supplementary Information

**Additional file 1.** Supplemnetary tables and figures.

## Data Availability

The results described here are a modeling study comprised from secondary data sources. No primary data collection was performed in relation to this work. All supporting data used for constructing the model is available in the public domain and has been cited or has been provided directly in the manuscript.

## References

[1] Deybach JP. Acute hepatic porphyria. 2009 [cited November 2020; Available from: https://www.orpha.net/consor/cgi-bin/OC_Exp.php?lng=EN&Expert=95157#:~:text=In%20the%20majority%20of%20European,between%2020%20to%2045%20years.

[2] Besur S (2014). Clinically important features of porphyrin and heme metabolism and the porphyrias. Metabolites.

[3] Wang B (2019). Acute hepatic porphyrias: review and recent progress. Hepatol Commun.

[4] Ramanujam VMS, Anderson KE. Porphyria diagnostics—part 1: a brief overview of the porphyrias. Curr Protoc Hum Genet. 2015;86(1):17.20. 1–17.20. 26. 10.1002/0471142905.hg1720s86 PMC4640448 26132003

[5] American Porphyria Foundation. Acute Hepatic Porphyria: Understanding Porphyria. 2020 [cited November 2020]. https://porphyriafoundation.org/purple-light-blog/acute-hepatic-porphyria-understanding-porphyria/.

[6] Gouya L (2020). EXPLORE: a prospective, multinational, natural history study of patients with acute hepatic porphyria with recurrent attacks. Hepatology.

[7] Naik H (2016). Experiences and concerns of patients with recurrent attacks of acute hepatic porphyria: a qualitative study. Mol Genet Metab.

[8] Schmitt C (2018). Recurrent attacks of acute hepatic porphyria: major role of the chronic inflammatory response in the liver. J Intern Med.

[9] Balwani M (2017). Acute hepatic porphyrias: recommendations for evaluation and long-term management. Hepatology.

[10] Elder G (2013). The incidence of inherited porphyrias in Europe. J Inherit Metab Dis.

[11] Puy H, Gouya L, Deybach J-C (2010). Porphyrias. The Lancet.

[12] Neeleman RA (2018). Medical and financial burden of acute intermittent porphyria. J Inherit Metab Dis.

[13] Auerbach AJ, Gokhale J, Kotlikoff LJ (1994). Generational accounting: a meaningful way to evaluate fiscal policy. J Econ Perspect.

[14] Mauskopf J (2018). Economic analysis of vaccination programs: an ISPOR good practices for outcomes research task force report. Value Health.

[15] Connolly MP (2017). The fiscal consequences attributed to changes in morbidity and mortality linked to investments in health care: a government perspective analytic framework. Value health.

[16] Statbel, Average gross monthly wages. 2018, STATBEL: Brussels.

[17] Statbel, Tax statistics on income subject to tax on natural persons by municipality of residence, B.a.t.r. Tax income, Editor. 2020, STATBEL: Brussels.

[18] OECD. Taxing Wages. Paris: OECD Publishing; 2019. 10.1787/tax_wages-2019-en.

[19] Statbel, Active (working and unemployed) population since 2017 based on the reformed Labour Force Survey, S. Belgium, Editor. 2019, STATBEL: Brussels.

[20] Statbel, Statbel (Directorate-general Statistics - Statistics Belgium) - EU-SILC 2018. 2020, Statbel.

[21] Statbel, Mortality tables and life expectancy, in STATBEL Life Tables. 2019.

[22] Statbel, Consumer price index. 2019, STATBEL: Brussels.

[23] Alnylam, Expert Consultation. 2020: Data on file.

[24] Samyn P. Vade Mecum; des données de la protection sociale en Belgique - partie statistique, S. sociale, Editor. 2019, Securite social: Bruxelles.

[25] Cleemput I, et al. Belgian guidelines for economic evaluations and budget impact analyses. 2012 [cited November 2020]. https://kce.fgov.be/sites/default/files/atoms/files/KCE_183_economic_evaluations_second_edition_Report_update.pdf.

[26] RIZIV Rijksinstituut voor ziekte- en invaliditeitsverzekering, NomenSoft Databank. 2020.

[27] invaliditeitsverzekering, R.R.v.z.-e. Geneesmiddelen. 2020 [cited November 2020]. https://www.riziv.fgov.be/nl/toepassingen/Paginas/farmaceutische-specialiteiten.aspx.

[28] EUNETHTA. Methos for health economic evalutions - A guideline based on current practices in Europe. 2015 [01 December 2020]. https://eunethta.eu/wp-content/uploads/2018/03/Methods_for_health_economic_evaluations.pdf.

[29] Pischik E, Kauppinen R (2015). An update of clinical management of acute intermittent porphyria. Appl Clin Genet.

[30] Balwani M (2020). Phase 3 trial of RNAi therapeutic givosiran for acute intermittent porphyria. N Engl J Med.

[31] NN. Average pension in Belgium versus abroad. 2013 [cited 2020 29 October 2020]. https://www.nn.be/nl/kapitalevragen/gemiddeld-pensioen-belgie-versus-buitenland#.

[32] NBB, N.B.o.B., Inflation and harmonised consumer price index, NBB.Stat, Editor. 2019.

[33] Belgium.be, Value Added Tax. government website, 2020.

[34] Anderson KE, Collins S. Open-label study of hemin for acute porphyria: clinical practice implications. Am J Med. 2006;119(9): 801 e19–24. 10.1016/j.amjmed.2006.05.026 16945618

[35] Andlin-Sobocki P (2005). Cost of disorders of the brain in Europe. Eur J Neurol.

[36] Breivik H (2013). The individual and societal burden of chronic pain in Europe: the case for strategic prioritisation and action to improve knowledge and availability of appropriate care. BMC Public Health.

[37] Nielens H, et al. Chronic low back pain. 2006 [cited November 2020]. https://kce.fgov.be/sites/default/files/atoms/files/d20061027371.pdf.

[38] Tack J (2019). Economic burden of moderate to severe irritable bowel syndrome with constipation in six European countries. BMC Gastroenterol.

[39] Liedgens H (2016). A burden of illness study for neuropathic pain in Europe. Clinicoecon Outcomes Res.

[40] McCrone P (2011). The economic costs of progressive supranuclear palsy and multiple system atrophy in France, Germany and the United Kingdom. PLoS ONE.

[41] Papanicolaou S (2005). Medical resource utilisation and cost of care for women seeking treatment for urinary incontinence in an outpatient setting. Examples from three countries participating in the PURE study. Maturitas.

[42] Gustavsson A (2011). Cost of disorders of the brain in Europe 2010. Eur Neuropsychopharmacol.

[43] Palmer AJ (2003). An economic evaluation of irbesartan in the treatment of patients with type 2 diabetes, hypertension and nephropathy: cost-effectiveness of Irbesartan in Diabetic Nephropathy Trial (IDNT) in the Belgian and French settings. Nephrol Dial Transplant.

[44] Onafhankelijke Ziekenfondsen. Health Forum. 2015 [cited March 2020]. https://www.mloz.be/nl/file/1588/download?token=DI2g6HtZ.

[45] Thein HH (2013). Health care costs associated with hepatocellular carcinoma: a population-based study. Hepatology.

[46] The Guardian. How much have I cost the NHS? 2016 [cited March 2020]. https://www.theguardian.com/society/ng-interactive/2016/feb/08/how-much-have-i-cost-the-nhs.

[47] Shei A (2015). Sources of prescription opioids among diagnosed opioid abusers. Curr Med Res Opin.

