# Supplementary material for: Estimating the broader fiscal consequences of acute hepatic porphyria (AHP) with recurrent attacks in Belgium using a public economic analytic framework
Source: Orphanet J Rare Dis. 2021 Aug 4;16:346. doi: 10.1186/s13023-021-01966-3 (PMC8336398; doi:10.1186/s13023-021-01966-3)
Supplement: Supplementary file 1 — Additional file 1. Supplemnetary tables and figures. [file 13023_2021_1966_MOESM1_ESM.docx]

**Additional File**

**Table S1 Costs for hemin-related adverse events**

| **Hemin-related AE** | **Incidence**  **[31]** | **Unit cost per event**  **[19]** |
| --- | --- | --- |
| Pyrexia | 0.0462 | €739.92 |
| Phlebitis/ISP | 0.0462 | €739.92 |
| Catheter-related complications | 0.0231 | €739.92 |
| Adverse drug reaction | 0.0231 | €739.92 |
| **Total** |  | **€102.45** |

**Table S2 Costs associated with co-morbidities in AHP-related attacks**

| **Comorbidity** | **Annual cost** | **Source** |
| --- | --- | --- |
| **Pain** |  |  |
| Headaches | € 330 | [32] |
| Chest pain | € 3,367 | [33] |
| Back pain | € 1,224 | [34, 35] |
| Abdomen pain | € 1,495 | [35] |
| Upper Extremities pain | € 3,367 | [33] |
| Lower Extremities pain | € 3,367 | [33] |
| Genitalia pain | € 3,367 | [33] |
| **Neurological comorbidity** |  |  |
| Paraesthesia | € 3,367 | Assumed equal to extremities pain [33] |
| Motor weakness | € 2,937 | Assumed equal to neuropathy [36] |
| Paralysis | € 106 | [37] |
| Urine incontinence | € 676 | [38] |
| Advanced Neuropathy | € 2,937 | Assumed equal to neuropathy [36] |
| **Psychiatric comorbidity** |  |  |
| Anxiety | € 942 | [32] |
| Depression | € 2,091 | [32] |
| Psychosis/Hallucinations | € 3,975 | [32] |
| Insomnia | € 604 | [39] |
| Suicidality | € 2,091 | Assumed equal to depression [32] |
| **Long-term conditions** |  |  |
| Hypertension | € 589 | [40] |
| Chronic kidney disease | € 35,270 | [41] |
| Hepatocellular carcinoma | € 14,908 | [42] |
| Hyponatremia | € 877 | Assumed equal to anaemia [43] |
| Epilepsy | € 2,995 | [32] |
| Anaemia | € 877 | [43] |
| Opioid addiction | € 1,784 | [44] |

**Table S3 Proportion of patients with comorbidities or long-term complications per health state**

| **Pain** | **Proportion of patients (%)** | | | |
| --- | --- | --- | --- | --- |
| Headaches | 36.4% | 36.4% | 29.2% | 13.2% |
| Chest pain | 9.1% | 9.1% | 4.2% | 1.9% |
| Back pain | 45.5% | 45.5% | 33.3% | 7.5% |
| Abdomen pain | 90.9% | 90.9% | 79.2% | 28.3% |
| Upper extremities pain | 36.4% | 36.4% | 25.0% | 3.8% |
| Lower extremities pain | 45.5% | 45.5% | 25.0% | 5.7% |
| Genitalia pain | 0.0% | 0.0% | 8.3% | 0.0% |
| **Neurological conditions** | Proportion of patients (%) | | | |
| Paraesthesia | 36.4% | 36.4% | 8.3% | 7.5% |
| Motor weakness | 45.5% | 45.5% | 20.8% | 7.5% |
| Paralysis | 9.1% | 9.1% | 20.8% | 1.9% |
| Urine incontinence | 0.0% | 0.0% | 4.2% | 0.0% |
| Advanced neuropathy | 27.3% | 27.3% | 20.8% | 0.0% |
| **Psychiatric disorders** | Proportion of patients (%) | | | |
| Anxiety | 45.5% | 45.5% | 20.8% | 5.7% |
| Depression | 36.4% | 36.4% | 12.5% | 9.4% |
| Psychosis/Hallucinations | 36.4% | 36.4% | 4.2% | 9.4% |
| Insomnia | 27.3% | 27.3% | 20.8% | 11.3% |
| Suicidality | 18.2% | 18.2% | 0.0% | 1.9% |
| **Long-term conditions** | Proportion of patients (%) | | | |
| Hypertension | 72.7% | 72.7% | 70.8% | 26.4% |
| Chronic kidney disease | 63.6% | 63.6% | 45.8% | 13.2% |
| Hepatocellular carcinoma | 9.1% | 9.1% | 8.3% | 1.9% |
| Hyponatremia | 72.7% | 72.7% | 37.5% | 0.0% |
| Epilepsy | 45.5% | 45.5% | 12.5% | 0.0% |
| Anaemia | 63.6% | 63.6% | 16.7% | 5.7% |
| Opioid addiction | 63.3% | 63.3% | 0.0% | 0.0% |

*AAR: Annualised attack rate*

**Figure 2 Impact of AHP on government transfers and taxes attributed to a person diagnosed at age 30 and experiencing 12 attacks per year for 10 years and unable to work discounted 3%**

**Figure 3 Impact of AHP on government transfers and taxes attributed to a person diagnosed at age 30 and experiencing 12 attacks per year for 10 years and returning to work discounted 3% (Scenario 3)**
